# Impact of educational intervention on the uptake of self-sampling for HPV test-based cervical cancer screening

**DOI:** 10.3389/fonc.2026.1810950

**Published:** 2026-04-22

**Authors:** Adeola Akintola, Eileen O. Dareng, Sally N. Adebamowo, ACCME Research Group as part of the H3Africa Consortium, C. A. Adebamowo, S. N. Adebamowo, MA. Famooto, A. Akintola, O. Adeyemo, R. Offiong, O. Olaniyan, A. Adebayo, S. Ologun, B. Alabi, P. Achara, R. A. Bakare, M. Odutola, O. Olawande, J. Okuma, G. Odonye, R. Adebiyi, P. Dakum, J. I. Erhunmwonsere, O. Owoade, T. Gbolahan, O. Ikwueme, Clement A. Adebamowo

**Affiliations:** 1 Division of Research, Center for Bioethics and Research, Ibadan, Nigeria; 2 Institute of Human Virology Nigeria, Abuja, FCT, Nigeria; 3 Department of Epidemiology and Public Health, and the Greenebaum Comprehensive Cancer Center, University of Maryland School of Medicine, Baltimore, MD, United States

**Keywords:** cervical cancer, education, HPV, Nigeria, screening, self-sampling

## Abstract

**Background:**

Self-sampling for HPV testing is increasingly adopted for cervical cancer screening globally, including in Sub-Saharan Africa. However, concerns remain regarding women’s willingness and ability to collect samples and the effectiveness of educational interventions. Although prior studies in Africa and Nigeria have examined acceptability and barriers, there is limited evidence on whether structured educational interventions can modify women’s attitudes toward HPV self-sampling in routine screening contexts. We evaluated the effect of a structured educational intervention on women’s attitudes toward self-sampling and, secondarily, explored baseline correlates of willingness to self-sample among Nigerian women.

**Methods:**

We conducted a single-group pre-post quasi-experimental study nested within the African Collaborative Center for Microbiome and Genomics (ACCME) prospective cohort study in central Nigeria. A total of 220 eligible women undergoing cervical cancer screening were enrolled. Baseline measurements were obtained prior to the intervention. The standardized educational intervention, delivered by trained research staff, included brochures, leaflets, an instructional video, verbal instructions, and hands-on familiarization with the Evalyn^®^ self-sampling brush. Participants subsequently performed self-sampling privately at participating screening facilities. Post-intervention measurements were collected immediately after the educational session and procedure. Samples were analyzed using DEIA/LIPA HPV assays.

**Results:**

Most participants were married (63.2%), belonged to the middle socioeconomic group (69.5%), had prior knowledge of cervical cancer (61.8%), and had never undergone screening (89.5%). At baseline, 91.8% were willing to self-sample. The intervention significantly improved mean attitude scores from 42.6 (SD 8.3) to 50.8 (SD 9.8) (p<0.001). Among women unwilling to self-sample, 50.0% (9/18) were in the lower SES group compared with 12.9% (26/202) among willing participants (p<0.001). In exploratory analyses, younger age (OR 0.95, 95% CI 0.90–1.00), cervical cancer knowledge (OR 1.42, 95% CI 1.00–1.99), middle SES (OR 3.69, 95% CI 1.07–12.66), and pre-intervention attitude (OR 0.89, 95% CI 0.81–0.99) were associated with willingness.

**Conclusions:**

A structured educational intervention significantly improved attitudes toward HPV self-sampling. Baseline willingness was high, and exploratory analysis indicate that younger age, better knowledge, and middle SES are associated with willingness. These findings support context-specific educational strategies to optimize HPV self-sampling uptake in Nigeria and similar low-resource settings.

## Background

The implementation of population based cervical cancer screening programs in high income countries (HIC) over the past few decades has significantly reduced the incidence and mortality of cervical cancer (1, 2). In contrast, the incidence and mortality in low- and middle-income countries (LMIC) remain high where it is still either the leading or second leading cause of cancer death among women (1, 2). This is due to several factors including high prevalence of HPV infection, failure of implementation of screening technologies like Pap smear that have worked in HIC, poorly resourced healthcare systems, lack of trained personnel, limited infrastructure, high cost, and high prevalence of co-morbidities such as HIV infection (3–5). Other reasons include healthcare access, geographic access, socio-cultural and religious beliefs, stigmatization and discrimination, low levels of awareness of cervical cancer and its risk factors, and health seeking behavior (4, 6, 7).

HPV-test based screening is now recommended by the WHO and other standards setting organizations for cervical cancer screening in different parts of the world, including in LMICs where other methods have not been effective (8–10). However in LMICs, the utilization and uptake of HPV-based screening remains sub-optimal more than a decade after their efficacy was demonstrated in randomized clinical trials (10). Several innovations have substantially reduced the cost, complexity, ease-of-use, accuracy, and turn-around time for HPV tests (11). But barriers including the complexity of HPV test based screening programs, limited number of trained health care providers, limited access to screening clinics, stigmatization, discrimination, modesty, spirituality, and lack of trust in the health care system continue to challenge HPV test based programs (12, 13).

Self-collection of samples for HPV tests may overcome some of the personal and healthcare system related barriers to cervical cancer screening (14). Trials comparing self to healthcare provider collection of samples for HPV test have found that both methods have at least similar efficacy or self-collection leads to higher rates of completion of cervical cancer screening (14, 15). Several educational interventions have been proposed to improve women’s attitudes, levels of acceptance, and uptake of self-collection for HPV testing (16, 17). The results of these educational interventions have varied across geographic location, sociocultural and religious beliefs (18–22). However, the remaining gap is not simply variation across studies.

Most prior studies in sub-Saharan Africa have described acceptability of self-sampling or compared self-collected with clinician-collected specimens, whereas fewer studies have prospectively measured whether a structured educational intervention changes women’s attitudes toward self-sampling within the same population. In Nigeria, barriers such as modesty, spirituality, and limited knowledge have been described, but evidence on whether a brief, clinic-delivered educational package can modify these concerns and improve willingness to self-sample among women presenting for screening remains limited (6, 7, 15). This information is needed to guide implementation of HPV self-sampling programs in routine screening settings. The primary objective of this study was to evaluate the effect of a structured educational intervention on women’s attitudes toward self-collection of samples for HPV testing. A secondary exploratory objective was to identify baseline correlates of willingness to self-sample among urban Nigerian women.

## Methods

This was a single-group pre-post quasi-experimental study nested within the African Collaborative Center for Microbiome and Genomics (ACCME) prospective cohort study of HPV infection and cervical cancer. In 2017, we recruited two hundred and twenty women who volunteered for cervical cancer screening as part of the African Collaborative Center for Microbiome and Genomics (ACCME) prospective cohort study of the HPV infection and cervical cancer at seven participating hospitals – National Hospital, Abuja; University of Abuja Teaching Hospital, Abuja; Garki hospital, Abuja; Wuse Hospital, Abuja; Asokoro District Hospital, Abuja; Kubwa general hospital, Abuja and Federal Medical Center, Keffi – in central Nigeria (23). We excluded women who were younger than 18 years or older than 65 years, with previous history of hysterectomy, pregnant, sexually naïve, with previous history of cervical intra-epithelial neoplasm (CIN) grade II or higher or cervical cancer, or women who were physically unable to self-sample.

### Sample size

To detect a pre-post difference in attitude scores following an educational intervention using McNemar’s test for correlated proportions, we calculated using McNemar’s test formula, which accounts for the paired (correlated) nature of pre- and post-intervention measurements. Using an expected difference in proportions of 12%, a power of 80%, a two-sided alpha of 0.05, and assuming a moderate degree of pre-post correlation (ρ = 0.2-0.3), the minimum required sample size ranged from approximately 197 to 262 participants. We enrolled 220 participants to meet this requirement.

### Educational intervention and self-sampling procedure

We administered a pre-intervention questionnaire in which we asked about the women’s knowledge and attitude to self-collection of samples for HPV test based cervical cancer screening. Participants were asked to respond to 8 questions on their attitude to self-sampling using a seven-point Likert scale with responses ranging from strongly disagree to strongly agree. The educational intervention was standardized and delivered during the same study visit by trained research staff. It included brochures, leaflets, an instructional video on cervical cancer screening and HPV self-sampling, a verbal step-by-step explanation of the procedure, demonstration of the Evalyn^®^ brush, an opportunity for participants to handle the device and ask questions, and clarification of common concerns regarding pain, safety, and infection. Participants were provided with instructions on use of Evalyn^®^ brush – a self-sample brush used in this study – and they were given Evalyn brushes to handle and become familiar with. Women then performed self-sampling privately in an office during the study visit at the participating screening clinics/hospitals. Samples were subsequently analyzed using DEIA/LIPA HPV tests.

### Measurements

We used the theory of planned behavior (TPB) to construct our post-intervention questionnaire based on our literature review, our previous qualitative studies on self-sampling, and discussions within our study team (15, 24). According to this theory, behavior is driven by three constructs – attitudes, subjective norms, and perceived behavioral control. For this study, “behavior” was defined as the self-collection of samples for HPV testing in cervical cancer screening. We used direct measurements e.g., by asking respondents about their overall attitude, and indirect measurements, e.g. by asking respondents about specific behavioral beliefs and outcome evaluations. Baseline measurements included socio-demographic characteristics, previous screening history, knowledge of cervical cancer, concerns about self-sampling, and pre-intervention attitude score. Post-intervention attitude and satisfaction were measured during the same visit immediately after the educational session and completion of the self-sampling procedure. We pre-tested our survey tools among twenty respondents to detect ambiguity, difficult to answer questions, appropriate length of the questionnaire, wording, and formatting. We used the outcome of the pre-test to revise our questionnaires. We re-coded scale items for negatively worded responses. We conducted item analysis to establish internal consistency of our questions and retained only those with internal consistency coefficients with Cronbach’s alpha > 0.6, which were included in the composite variable for knowledge, subjective norms, perceived behavioral control, and attitude. Question items with low internal consistency were excluded from the composite score. Participants were asked to rate their satisfaction with the self-sampling process on a seven-point Likert scale ranging from extremely satisfied to extremely dissatisfied. We used the wealth index based on the principal component analysis of data on household assets as described by Filmer and Pritchett to compute the socioeconomic status of the participants (25). For the secondary exploratory analysis, willingness to self-sample was defined as an affirmative response to the baseline question asking whether the participant would be willing to use self-sampling for HPV testing as a screening method. This variable reflected screening preference/intention and not willingness to participate in the study; all women included had already consented to enroll.

### Statistical analysis

We summarized categorical variables with counts and percentages and continuous variables with means and standard deviations. We compared the characteristics of women who were willing and unwilling to self-sample at baseline using chi-square tests or Fisher’s exact tests for categorical variables and Student’s t-test for age. The primary analysis compared the mean pre- and post-intervention attitude scores using a paired t-test. Secondary exploratory analyses used logistic regression to estimate odds ratios (ORs) and 95% confidence intervals (CIs) for baseline correlates of willingness to self-sample. Statistical significance was defined as p<0.05.

## Results

### Participants characteristics

Some 220 women participated in this study. Their mean (SD) age was 33.5 (10.2) years. Most participants (139/220, 63.2%) were married. About half of the participants (112/220, 50.9%) were formally employed while 49.1% were not. Most (153/220, 69.5%) of the women were in the middle socio-economic status (SES) category, 14.5% (32/220) were in the upper SES while 15.9% (35/220) were in the lower SES. Most of the participants (132/220, 60.0%) have had more than 12 years of education while 40.0% (88/220) have had 12 years of education or less. (**Table 1**) Although 136/220 (61.8%) reported prior knowledge of cervical cancer, only 10.5% (23/220) had ever previously screened for cervical cancer (**Table 1**).

**Table 1:** Sociodemographic characteristics of participants by baseline willingness to self-sample, Abuja, Nigeria, 2017.

Characteristic	Unwilling n = 18	Willing n = 202	Total n = 220	p-value
Age, mean (SD)	37.9 (9.0)	33.1 (10.2)	33.5 (10.2)	0.05
Not married	6 (33.3)	75 (37.1)	81 (36.8)	0.75
Married	12 (66.7)	127 (62.9)	139 (63.2)	
Currently working	9 (50.0)	103 (51.0)	112 (50.9)	0.94
Not working	9 (50.0)	99 (49.0)	108 (49.1)	
<=12 years of education	14 (77.8)	74 (36.6)	88 (40.0)	0.001
>12 years of education	4 (22.2)	128 (63.4)	132 (60.0)	
Lower SES	9 (50.0)	26 (12.9)	35 (15.9)	<0.001
Middle SES	6 (33.3)	147 (72.8)	153 (69.5)	
Upper SES	3 (16.7)	29 (14.4)	32 (14.5)	
Knowledge of cervical cancer: Yes	4 (22.2)	132 (65.3)	136 (61.8)	0.001
Knowledge of cervical cancer: No	14 (77.8)	70 (34.7)	84 (38.2)	
History of cervical cancer screening: Yes	0 (0.0)	23 (11.4)	23 (10.5)	0.23
History of cervical cancer screening: No	18 (100.0)	179 (88.6)	197 (89.5)	

Values are n (%) unless otherwise stated. p-values are from chi-square tests or Fisher’s exact test, except age which was compared using Student’s t-test.

### Baseline willingness to self-sample

At baseline, 202/220 (91.8%), reported that they were willing to self-collect samples for HPV testing whereas 8.2% (18/220) reported that they were not. Women who were willing to self-collect samples were younger (mean (SD) = 33.1 (10.2) years) than those who were not willing to self-collect (37.9 (9.0), *p* = 0.05). Educational attainment was higher among women who were willing to self-sample than among those who were unwilling (63.4%, 128/202 vs 22.2%, 4/18 with >12 years of education; p=0.001). Among the 18 women who were unwilling to self-sample, half (50.0%, 9/18) were from the lower SES group, compared with 12.9% (26/202) of women who were willing to self-sample (p<0.001). Most women who were willing to self-sample were in the middle SES category (72.8%, 147/202). Most women (144/220, 65.4%) had never heard of HPV as a cause of cervical cancer. Knowledge about cervical cancer was more common among women who were willing to self-sample (65.3%, 132/202) than among those who were unwilling (22.2%, 4/18; p=0.001). Women who were willing to self-sample were also more likely to have knowledge of cervical cancer symptoms (65.3% vs 38.9%, p=0.04) and to know that family history of cervical cancer (21.8% vs 0.0%, p=0.03) and multiple sexual partners (36.6% vs 0.0%, p<0.01) are risk factors for cervical cancer (**Tables 1**, **2**). In exploratory logistic regression analyses, age (OR = 0.95, 95% CI 0.90-1.00), knowledge of cervical cancer (OR = 1.42, 95% CI 1.00-1.99), middle SES (OR = 3.69, 95% CI 1.07-12.66), and pre-intervention attitude (OR = 0.89, 95% CI 0.81-0.99) were associated with willingness to self-sample (**Table 3**).

**Table 2:** Knowledge of HPV and cervical cancer risk factors by baseline willingness to self-sample, Abuja, Nigeria, 2017.

Knowledge item (yes response)	Unwilling n = 18	Willing n = 202	Total n = 220	p-value
Ever heard of HPV as a cause of cervical cancer	1 (5.6)	75 (37.1)	76 (34.6)	<0.01
Ever heard of cervical cancer screening	5 (27.8)	120 (59.4)	125 (56.8)	0.01
Knows family history is a risk factor	0 (0.0)	44 (21.8)	44 (20.0)	0.03
Knows multiple sexual partners are a risk factor	0 (0.0)	74 (36.6)	74 (33.6)	<0.01
Knows smoking is a risk factor	3 (16.7)	22 (10.9)	25 (11.4)	0.44
Knows obesity is a risk factor	0 (0.0)	7 (3.5)	7 (3.2)	1.00
Knows HIV infection is a risk factor	1 (5.6)	18 (8.9)	19 (8.6)	1.00
Knows inserting foreign objects into the vagina is a risk factor	2 (11.1)	31 (15.4)	33 (15.0)	1.00
Knows douching is a risk factor	2 (11.1)	45 (22.3)	47 (21.4)	0.38

Values are n (%).

**Table 3:** Factors associated with baseline willingness to self-collect samples for cervical cancer screening.

Variable	OR	p-value	95% CI
Age	0.95	0.07	0.90 to 1.00
Knowledge of cervical cancer	1.42	0.05	1.00 to 1.99
SES: Upper	2.81	0.20	0.57 to 13.71
SES: Middle	3.69	0.04	1.07 to 12.66
Pre-intervention attitude	0.89	0.03	0.81 to 0.99

Odds ratios (ORs) and 95% confidence intervals (CIs) are from the logistic regression model.

### Effect of educational intervention on attitude

The mean (SD) composite score for attitude to self-collection was 42.6 (8.3) before the educational intervention and 50.8 (9.8) after the intervention (paired p<0.001) (**Table 4**).

**Table 4:** Effect of educational intervention on attitude toward self-collection.

Time point	n	Mean score	SD	95% CI for mean	p-value
Pre-intervention	220	42.6	8.3	41.5 to 43.7	<0.001
Post-intervention	220	50.8	9.8	49.5 to 52.1	

p-value is from the paired t-test comparing pre- and post-intervention attitude scores.

### Knowledge, experience, and concerns related to self-sampling

The use of tampons was uncommon, with only 16/220 (7.3%) reporting ever having used a tampon, all of whom were willing to self-sample. A total of 66/220 (30.0%) reported having inserted foreign materials into the vagina for purposes other than managing menstruation (**Table 5**). When participants were asked about concerns they might have about self-collecting samples, 95/220 (43.2%) were concerned about their ability to do it properly, 77/220 (35.0%) were concerned about the safety of the procedure, 56/220 (25.5%) were concerned that self-collection might hurt, 41/220 (18.6%) were concerned that it might cause infection or irritation, and 19/220 (8.6%) were uncomfortable touching their genital area. There was no statistically significant difference in these specific concerns between women who were willing and unwilling to self-sample at baseline (**Table 6**).

**Table 5:** Knowledge of symptoms and prior vaginal insertion experiences by baseline willingness to self-sample, Abuja, Nigeria, 2017.

Item	Unwilling n = 18	Willing n = 202	Total n = 220	p-value
Has knowledge of symptoms of cervical cancer	7 (38.9)	132 (65.3)	139 (63.2)	0.04
Ever used a tampon	0 (0.0)	16 (7.9)	16 (7.3)	1.00
Has inserted foreign materials into the vagina for purposes other than managing menstruation	3 (16.7)	63 (31.2)	66 (30.0)	0.28

Values are n (%).

**Table 6:** Concerns about self-sampling by baseline willingness to self-sample, Abuja, Nigeria, 2017.

Concern (yes response)	Unwilling n = 18	Willing n = 202	Total n = 220	p-value
Concerned about ability to do it properly	5 (27.8)	90 (44.6)	95 (43.2)	0.17
Concerned about safety of the procedure	7 (38.9)	70 (34.7)	77 (35.0)	0.72
Concerned that self-collection may hurt	2 (11.1)	54 (26.7)	56 (25.5)	0.17
Concerned that self-collection may cause infection or irritation	2 (11.1)	39 (19.3)	41 (18.6)	0.54
Uncomfortable touching the genital area	3 (16.7)	16 (7.9)	19 (8.6)	0.19
Other reasons	0 (0.0)	16 (7.9)	16 (7.3)	0.37

Values are n (%).

## Discussion

HPV DNA self-sampling has emerged as a critical strategy in secondary prevention of cervical cancer in regions where HPV vaccination is low or non-existing. Despite its wide acceptance and deployment in high income countries, its adoption, access and penetration in low- and middle-income countries remains limited. In this study, a structured educational intervention significantly improved attitudes toward HPV self-sampling among women enrolled in cervical cancer screening clinics in central Nigeria. This finding addresses an implementation question that is distinct from simple acceptability estimates: whether a brief clinic-delivered educational package can shift attitudes in a setting where self-sampling is not yet widely established.

More than half of the participants reported prior knowledge of cervical cancer, aligning with findings from studies in the UK (26) and Cameroon (18). However, this contrasts with research from Kenya (27), Ghana (28), DRC (29) and Nigeria (15) where lower levels of knowledge were observed. Notably, women unwilling to self-sample demonstrated limited understanding of cervical cancer symptoms and risk factors (e.g., multiple sexual partners, family history, HPV infection). This knowledge gap likely contributed to their reluctance, underscoring the need for targeted education to improve awareness and screening uptake among women of reproductive age.

The majority of participants expressed willingness to use self-sampling for cervical cancer screening, consistent with findings from studies in Africa (18, 30–32) and Asia (33) and Canada (34). Our results showed higher willingness rates compared to studies in Cameroon (21), Tanzania and Kenya (35) possibly due to differences in participant recruitment settings, educational levels, perceptions of healthcare workers, or the sampling device use in this study. Because these willingness analyses were secondary and exploratory, they should be interpreted as hypothesis-generating rather than as the primary endpoint of the study.

Despite high willingness, participants raised concerns about the procedure, including doubts about their ability to self-sample correctly, safety, pain, infection risk, and discomfort with touching their genitalia. These concerns mirror findings from studies in South Africa (36), Switzerland (37), Bangladesh (38), Tanzania (30, 39) where similar reservations were reported by women who preferred clinician-collected samples. Addressing these concerns is critical to improving the acceptability of self-sampling.

The educational intervention significantly improved participants’ attitude scores, corroborating findings from studies in Cameroon (18), Malaysia (33) and US (40). Unlike some prior research, our study measured attitudes both before and after the intervention, providing robust evidence of its positive effect. This aligns with the Theory of Planned Behavior (TPB), which posits that attitude is a key determinant of behavioral intention (41).

Age, knowledge of cervical cancer, and pre-intervention attitude were independent predictors of willingness to self-sample. This is keeping in line with findings from other studies that have found age, knowledge as predictors of willingness to self-collect (15, 18). The inverse association between the pre-intervention attitude score and willingness should be interpreted cautiously because the baseline composite score included reverse-coded items and may reflect residual measurement complexity or unmeasured preference for clinician-collected sampling. This association therefore requires confirmation in larger studies.

Younger women were more willing to self-collect, contrasting with studies that found higher acceptance among older women (18, 34). One way to argue this finding may be the early exposure of women to information online which gives them early access than older population. Younger people tend to focus more on issues affecting their age group than older people who are more preoccupied with taking care of the home front. Also, we found that women with greater attitude score pre-intervention are less likely to be willing to self-collect sample. Some of the reasons for this may be that those women had earlier been exposed to physician assisted sample collection and are finding it hard to switch to other method. It could also be that they trust their healthcare providers (21, 32). Secondly, it could mean that some of the women had concerns with self-sampling and the introduction of the educational intervention did not erase their concerns.

The strengths of this study include the pre-post intervention design, use of the TPB framework, and measurement of attitudes before and after the intervention while the limitations include the small number of women who were unwilling to self-sample, and the inability to assess perceived behavioral control. These limitations reduce the precision and generalizability of subgroup analyses and suggest that findings from the exploratory willingness models should be interpreted cautiously. Future research should expand participant recruitment, incorporate additional reproductive variables, and explore attitudes toward different self-sampling devices and methods. Addressing perceived behavioral control and other TPB constructs could further elucidate factors influencing screening behavior.

In conclusion, a structured educational intervention improved attitudes toward HPV DNA self-sampling among women in central Nigeria. High baseline willingness and the observed associations with age, cervical cancer knowledge, and socioeconomic status suggest that implementation of self-sampling programs should combine accessible devices with culturally and contextually tailored education.

## Data Availability

The raw data supporting the conclusions of this article will be made available by the authors, without undue reservation.

## Group members of ACCME Research Group as part of the H3Africa Consortium

Adebamowo C. A., MD, ScD Department of Epidemiology and Public Health, and the Greenebaum Comprehensive Cancer Center, University of Maryland School of Medicine, Baltimore, Maryland, 21201, USA; Center for Bioethics and Research, Ibadan, Nigeria; Institute of Human Virology Nigeria, Abuja, Nigeria, Adebamowo S. N., MD, MSc, ScD Department of Epidemiology and Public Health, and the Greenebaum Comprehensive Cancer Center, University of Maryland School of Medicine, Baltimore, Maryland, 21201, USA; Center for Bioethics and Research, Ibadan, Nigeria, Famooto MA., Sc Institute of Human Virology Nigeria, Abuja, Nigeria, Akintola A. MPH Center for Bioethics and Research, Ibadan, Nigeria, Adeyemo O., MSc Center for Bioethics and Research, Ibadan, Nigeria, Offiong R., MD University of Abuja Teaching Hospital, Gwagwalada, Abuja, Nigeria, Olaniyan O., MD National Hospital Abuja, Abuja, Nigeria, Obende K., MD Garki Hospital Abuja, Abuja, Nigeria, Adebayo A., MD Asokoro District Hospital, Abuja, Nigeria Ologun S., MD Kubwa General Hospital Abuja, Nigeria, Alabi B., MD Wuse General Hospital, Abuja, Nigeria Achara P., MD Federal Medical Center, Keffi, Nigeria, Bakare R. A., MD University of Ibadan, Ibadan, Nigeria, Odutola M. MD Institute of Human Virology Nigeria, Abuja, Nigeria, Olawande O., MPH Institute of Human Virology Nigeria, Abuja, Nigeria, Okuma J., MPH Institute of Human Virology Nigeria, Abuja, Nigeria, Odonye G., BSc Institute of Human Virology Nigeria, Abuja, Nigeria, Adebiyi R., BSc Institute of Human Virology Nigeria, Abuja, Nigeria, Dakum P., MD Institute of Human Virology Nigeria, Abuja, Nigeria, Erhunmwonsere J. I., BSNc, RN, RM Institute of Human Virology Nigeria, Abuja, Nigeria, Owoade O., BSc, MSc Institute of Human Virology Nigeria, Abuja, Nigeria, Gbolahan T., BSc Institute of Human Virology Nigeria, Abuja, Nigeria, Ikwueme O., MPH Institute of Human Virology Nigeria, Abuja, Nigeria.
